# The Feasibility and Tolerability of Medium Chain Triglycerides in Women with a Catamenial Seizure Pattern on the Modified Atkins Diet

**DOI:** 10.3390/nu13072261

**Published:** 2021-06-30

**Authors:** Elizabeth A. Felton, Bobbie J. Henry-Barron, Amanda K. Jan, Abigail Shegelman, Kelly Faltersack, Diane Vizthum, Mackenzie C. Cervenka

**Affiliations:** 1 Department of Neurology, University of Wisconsin School of Medicine and Public Health, Madison, WI 53705, USA; 2 Institute for Clinical and Translational Research, Johns Hopkins University School of Medicine, Baltimore, MD 21202, USA; bjhenry@jhu.edu (B.J.H.-B.); dblahut1@jhmi.edu (D.V.); 3 Department of Neurology, Johns Hopkins University School of Medicine, Baltimore, MD 21287, USA; amanda@thejans.net (A.K.J.); ashegel1@jhu.edu (A.S.); mcerven1@jhmi.edu (M.C.C.); 4 Department of Clinical Nutrition Services, UW Health, Madison, WI 53792, USA; kfaltersack@uwhealth.org

**Keywords:** epilepsy, seizure, ketogenic diet therapy, catamenial, medium chain triglyceride (MCT)

## Abstract

Ketogenic diet therapy (KDT), particularly modified Atkins diet (MAD), is increasingly recognized as a treatment for adults with epilepsy. Women with epilepsy (WWE) comprise 50% of people with epilepsy and approximately one in three have catamenial epilepsy. The purpose of this study was to determine whether adding a medium chain triglyceride emulsion to MAD to target catamenial seizures was feasible and well-tolerated. This was a prospective two-center study of pre-menopausal WWE with a catamenial seizure pattern on MAD. After a 1-month baseline interval with no changes in treatment, participants consumed betaquik^®^ (Vitaflo International Ltd.) for 10 days each menstrual cycle starting 2 days prior to and encompassing the primary catamenial seizure pattern for five cycles. Participants recorded seizures, ketones, and menses, and completed surveys measuring tolerability. Sixteen women aged 20–50 years (mean 32) were enrolled and 13 (81.2%) completed the study. There was 100% adherence for consuming betaquik^®^ in the women who completed the study and overall intervention adherence rate including the participants that dropped out was 81.2%. The most common side effects attributed to MAD alone prior to starting betaquik^®^ were constipation and nausea, whereas abdominal pain, diarrhea, and nausea were reported after adding betaquik^®^. The high adherence rate and acceptable tolerability of betaquik^®^ shows feasibility for future studies evaluating KDT-based treatments for catamenial seizures.

## 1. Introduction

Ketogenic diet therapy (KDT) is increasingly recognized as effective for adults with epilepsy [1,2,3,4,5,6,7,8,9]. KDTs induce fat metabolism and ketone body production by consuming a high fat, low carbohydrate, adequate protein diet [9]. The modified Atkins diet (MAD) is a KDT most often used in adults and is typically composed of a 20 g/day limit in net carbohydrates (total carbohydrate minus fiber) with liberal fat and moderate protein intake [10]. MAD is a less restrictive diet compared to the classic ketogenic diet, which is prescribed as a ratio of grams of fat to grams of carbohydrate and protein combined (e.g., 3:1 or 4:1 ratio). The MAD does not have a prescribed macronutrient ratio, but people who follow the MAD are typically able to get into the state of ketosis.

Despite its known efficacy, there are large gaps in knowledge regarding use of KDTs in adults. These include KDTs’ effect on the menstrual cycle in women with epilepsy and how to best target seizures that correlate with the menstrual cycle. Women make up 50% of people with epilepsy and about one third have a pattern of seizures that correlates with their menstrual cycle, referred to as catamenial epilepsy. Three patterns have been identified—C1 (perimenstrual), C2 (periovulatory), and C3 (luteal) [11,12]. The hormonal fluctuations implicated in catamenial epilepsy include the surge of estrogen on day 13 of the menstrual cycle (day 1 is the first day of menses) and the rapid decline of progesterone and then estrogen on days 26–28. Progesterone withdrawal in particular has been implicated as a trigger for catamenial seizures [13,14]. There are many proposed pharmacologic strategies for treating women with catamenial epilepsy [13,15,16]. However, catamenial epilepsy is typically difficult to manage. Women with drug-resistant catamenial epilepsy may choose to start a KDT such as MAD, and diet modifications could be made to target catamenial seizure patterns.

Although not extensively studied, reports describe menstrual changes in women on KDTs, one dating back to 1930 where 12 of 56 women (21%) observed on the classic ketogenic diet had amenorrhea, and this was proposed to be due to hormonal changes or vitamin deficiency [17]. Another report in 2016 noted that only 2 of 85 women (2%) of reproductive age (18–49 years) reported amenorrhea on MAD or KD, although retrospectively, it may have been an underestimate [18]. We have observed at the Johns Hopkins Adult Diet Epilepsy Center (AEDC) and the UW Health Adult Epilepsy Dietary Therapy Clinic (AEDTC) that despite an overall reduction in seizures, some women on MAD continue to have breakthrough seizures in a catamenial pattern [19,20]. About half had a catamenial pattern prior to starting MAD, and this pattern persisted. The other half did not have a defined pattern prior to starting MAD. Although typically reduced, seizures that continued occurred in a catamenial pattern. The potential interactions between KDTs and the menstrual cycle resulting in these observed changes are not yet understood. However, they may be due to difficulty in maintaining ketosis around ovulation or menses secondary to either a hormone-mediated mechanism or not adhering as strictly to MAD during that time (some women on MAD have described “carbohydrate cravings” during menses). Finally, carbohydrate restriction and weight loss may impact hormone levels, altering the menstrual cycle and potentially seizure control [21].

For women on MAD with catamenial seizures, we proposed to increase the fat content of the diet with medium chain triglyceride (MCT) oil, during the phase of their menstrual cycle when these seizures were observed. MCTs are more ketogenic than long-chain triglycerides (LCT), and MCT oil is often recommended as a supplement to the MAD [9,22]. MCT oil was introduced in the same way as prior pharmaceutical interventions for catamenial epilepsy beginning 2 days prior to the time point in their menstrual cycle, when catamenial seizures are normally observed, and continuing it for a total of 10 days [16]. We hypothesized that patients already adherent on MAD would tolerate and be adherent with the intervention.

## 2. Materials and Methods

### 2.1. Study Design and Participants

This was a prospective, two-center study of pre-menopausal women with a history of drug-resistant epilepsy on MAD for at least 3 months who had a catamenial seizure pattern defined by Herzog criteria [11]. The study was conducted to determine if adding an MCT-based medical food product (betaquik^®^, Vitaflo International Ltd., Liverpool, UK) was feasible and well-tolerated.

Catamenial seizure patterns were determined by analyzing calendars where women annotated dates and quantity of seizures and menses onset. The presence or absence of a catamenial pattern was determined by calculating a ratio based on average daily seizure frequency during the target phase compared to the others. The ratios used in this study were previously validated [11].

The study was approved by the Johns Hopkins and University of Wisconsin-Madison Institutional Review Boards and reported in ClinicalTrials.gov (NCT02426047). Written informed consent was obtained from all participants or a legally authorized representative. Participants were seen at the Johns Hopkins Hospital AEDC or UW AEDTC between March 2015 and December 2019.

Inclusion criteria were pre-menopausal women, age ≥18 years, already adherent on a 20-g net carbohydrate per day limit MAD [18] for at least 3 months, determined to have an established catamenial seizure pattern (by Herzog criteria [11]) during at least 2 of the past 3 months.

Exclusion criteria included men; being unwilling to restrict carbohydrates; significantly underweight (BMI ≤ 18.5); pregnant or anticipating pregnancy within 6 months; diagnosed with kidney disease; severe hypercholesterolemia (>300 mg/dL); hypertriglyceridemia (>200 mg/dL); metabolic or mitochondrial disorder for which a KDT is contraindicated; lactose intolerance or milk allergy; women with an aversion to liquids or inability to eat solid food; prior use of betaquik^®^ at any time for any duration; planning to adjust antiseizure treatment(s) or hormonal contraception within the next 6 months; and women who were menopausal, peri-menopausal, or taking hormonal contraceptive continuously to avoid menstruation, or already using another KDT supplement (e.g., medium chain triglyceride oil, ketogenic medical food, exogenous ketones) for the purpose of stimulating ketosis (unless the patient was using one daily and was willing to continue doing so for the duration of this study).

### 2.2. Study Procedures

Women who qualified were consented and enrolled during a routine follow-up outpatient clinic appointment. Participants were counseled to leave all antiseizure drugs and hormonal contraceptives unchanged throughout the 6-month study unless medically necessary and to inform the study team immediately of any changes, plans to become pregnant, or discovered pregnancy.

During a 1-month baseline period, participants were asked to keep a seizure and menstrual cycle calendar; check and record daily urine ketones and weekly weight; and complete a baseline 3-day food record. To evaluate for ovulatory cycles, during the baseline month, a progesterone level was drawn on day 22–24 of the menstrual cycle (with day 1 = first day of menses) and ideally within 3–11 days of the start of the next menstrual cycle. Participants who were not ovulating were still included in the study since women with irregular menses and anovulatory cycles can also have catamenial seizure patterns.

After the 1-month baseline interval, study participants remained on MAD and added betaquik^®^ for 10 days per month for the final 5 months of the 6-month study. Study participants were contacted each month and given specific instructions for which 10-day period to consume betaquik^®^. They were asked to note on provided calendars which days they consumed it and if there were any days they missed for any reason. To introduce the intervention gradually and avoid intolerance, participants were instructed to consume ¼ carton (62 mL, 12.5 g of MCT fat) of betaquik^®^ on the first day and ½ carton (125 mL, 25 g of MCT fat) for the remaining 9 days. The exact days to consume betaquik^®^ were determined by the individual catamenial seizure pattern when reviewing the seizure calendar from the month prior. The required amount of betaquik^®^ could be consumed on the target days in any manner (i.e., drink all at once or smaller amounts throughout the day). They were also given sample recipes for using betaquik^®^ if they chose to consume it with food. A 24-h food record was obtained via phone by the dietitian during one of the days they consumed betaquik^®^ each month.

Participants were provided test strips to measure urine ketones (acetoacetate) and were also asked to check and record ketones daily during the baseline month, during the 10 days they took betaquik^®^, and twice weekly for the rest of the month. Ketones were recorded as: negative, trace (5 mg/dL), small (15 mg/dL), moderate (40 mg/dL), large (80 mg/dL) or large plus (160 mg/dL).

Outpatient clinic visits occurred at enrollment and at the 3-month and 6-month time point during the study. At these visits, the participants met with both the site principal investigator and ketogenic dietitian. Basic anthropometrics, including height and weight, were recorded at each visit. Tolerance and menstrual cycle surveys were filled out at each visit as well. A pre-betaquik^®^ survey asked participants to rate the convenience, ease of use, and taste of foods consumed on MAD, and to report any symptoms they attributed to MAD. An on-betaquik^®^ survey asked participants to rate the convenience, ease of use, and taste of the betaquik^®^ added to MAD and to report any symptoms they believed were due to MAD or betaquik^®^. Symptoms queried were abdominal pain, constipation, diarrhea, nausea, vomiting, fatigue, hunger, problems with texture/mouthfeel, problems with taste, and there was also a space to include unsolicited symptoms.

Participants exited the trial if they withdrew from the study for any reason. Participants were considered study failures if they did not complete the 10-day per month use of betaquik^®^ for at least 2 of the 5 months or if they did not adhere to the 20-g of net carbohydrates per day MAD. When the study reached completion, participants could elect to continue use of betaquik^®^ if desired or to replace betaquik^®^ with an equivalent dose of commercial MCT oil, if preferred.

### 2.3. Outcome Measures

The primary outcome measure was adherence with betaquik^®^ compared to published adherence with MAD and medium chain triglyceride diets to demonstrate feasibility. Adherence was measured by the percent of time the participant drank betaquik^®^ as instructed (participants were instructed to take it for 10 days per month and annotate when taken on the calendar). The participant was defined as adherent if they drank the required amount (either ¼ or ½ carton) on more than 80% of the prescribed days.

A secondary outcome measure was tolerability of betaquik^®^ based on a 10-point tolerance scale. Tolerance was measured by surveys given to the participants before and after starting betaquik^®^.

### 2.4. Power Analysis

Prior studies of MAD [23,24,25] and medium chain triglyceride diets [26] have shown an approximately 55–60% adherence rate at 3–8 months (either reported as such or calculated using the number of participants who dropped out of the study). To be included in the study, participants needed to demonstrate adherence on MAD prior to enrollment. We hypothesized that MAD plus betaquik^®^ would lead to an 85% adherence rate compared to a 55% adherence rate of a MAD or MCT diet alone. Based on this, we calculated a sample size of 19 to achieve 80% power and an alpha level of 0.05. Our goal enrollment was 20 participants, taking into account potential attrition and protocol violations.

### 2.5. Statistical Analysis

Descriptive statistics were used to present baseline characteristics. Proportions were calculated for all categorical variables and means or medians, standard deviations, and ranges were calculated for all continuous variables. Differences between participant ratings for MAD and MAD plus the intervention were assessed using a paired samples *t*-test.

## 3. Results

### 3.1. Participant Demographics

Between March 2015 and May 2019, 16 women aged 20–50 years (mean 32, SD 8.0) who were already on MAD for at least 3 months through the Johns Hopkins AEDC or UW Health AEDTC were enrolled (Table 1). Age of seizure onset ranged from 1–41 years (mean 10.9, SD 10.8). Duration of MAD use prior to study enrollment ranged from 0.5–6 years (mean 1.9, SD 1.7).

Of the 16 participants who enrolled, 13 (81%) completed the study. Three participants dropped out, two of these were due to gastrointestinal (GI) side effects deemed likely related to the study intervention, betaquik^®^. A third participant had both GI side effects and missed menses after the first month of the study. The reported GI side effects included nausea and abdominal pain, and none required medical attention. The participants who dropped out were older with an average age of 42.7 years, compared to the average age of participants who completed the study at 30.6 years.

### 3.2. Catamenial Pattern

Women with a C1 (perimenstrual), C2 (periovulatory) or C3 (luteal) pattern for at least 2 of the prior 3 months while on KDT were invited to enroll in the study. At baseline, *n* = 15 (93.8%) had primarily a C3 catamenial pattern (some women had more than one pattern). During the intervention months, the majority (75%) of participants had 1 or more months where there were seizures, but without a catamenial pattern, and *n* = 3 of the 13 (23.1%) who completed the study had no catamenial pattern for the last 2 months of the study. A total of *n* = 6 (37.5%) had at least 1 month with no seizures during the study.

Fourteen participants obtained a serum progesterone level (one was never obtained and one had the incorrect laboratory study measured), and the mean level was 8.0 ng/mL, range 0.6–17.1 ng/mL, SD 4.6 ng/mL. Of the 14 participants, only 5 (35.7%) had a value of 10 ng/mL or above, which is an indicator of ovulation. The average number of days to the next menstrual cycle was 7 days, with a range of 3–13 days. Two of the 14 participants had progesterone levels measured outside the ideal 3–11 day window prior to menses onset. As noted above, a majority of women in the study had C3 catamenial pattern, where seizures are increased during the entire luteal phase in anovulatory cycles, which typically results in irregular menses.

### 3.3. Ketones

At month 1 (baseline—no intervention), *n* = 6 (37.5%) of participants recorded ketones as requested, while *n* = 5 (31.3%) recorded ketones once or more, but not per protocol with the full data requested. During month 2 (first month with the addition of betaquik^®^), *n* = 12 (75%) recorded ketones and *n* = 3 (18.8%) recorded ketones once or more, but not per protocol. During month 5 when 13 participants remained, *n* = 9 (69.2%) recorded ketones and *n* = 3 (23.1%) recorded ketones once or more, but not per protocol.

During months with enough ketone data available to analyze, levels of each participant were evaluated to determine whether urine ketone values were similar, higher, or lower during the 10-day timeframe betaquik^®^ was added to KDT, compared to the rest of the month. Average ketone levels were never lower during the betaquik^®^ days. Evaluating across the entire study, *n* = 11 (68.8%) of all participants had higher ketones during at least one of the 5 months betaquik^®^ was consumed, and *n* = 5 (31.3%) had higher ketones levels for at least 3 of the 5 months.

### 3.4. Seizures

This study was not designed to evaluate efficacy of betaquik^®^ as a primary outcome. However, the baseline (non-intervention) month was compared with the 5 months the participants received the intervention. Of the 16 participants, *n* = 6 (37.5%) had at least one of the 5 months during the betaquik^®^ intervention with no seizures, although two of these participants also had no seizures during their baseline MAD only month (in addition to at least one other month during the intervention). There were *n* = 7 (43.7%) who had fewer seizures on average, including *n* = 4 (25%) with a 50% or greater seizure reduction, during the intervention months compared to the baseline month. One participant had six seizures during the baseline month and six total seizures during the 5 intervention months combined.

### 3.5. Adherence

Of the 13 participants who completed the study, there was 100% adherence (consuming the required amount of betaquik^®^ on more than 80% of the prescribed days.) Of the 3 participants who dropped out of the study, there was also 100% adherence until the point of study exit. The overall intervention adherence rate through study completion was 81.2%.

### 3.6. Tolerance

Participants were asked to rate the convenience, ease of use, and taste of MAD (at enrollment) and MAD + betaquik^®^ (3-month and 6-month time points) on a 10-point Likert scale (Table 2). There was no significant difference in ease of use or taste between time points. There was a significant increase in the convenience rating at both the 3-month and 6-month time points (MAD + betaquik^®^) compared to baseline (MAD only).

Participants were asked to indicate if they experienced any symptoms that they attributed to MAD (at baseline and at the 3- and 6-month time points) or to betaquik^®^ (at the 3- and 6-month time points). At the 3-month time point, there were 14 surveys completed due to one participant dropping out and one not completing the 3-month survey. At the 6-month time point there were 13 due to the 3 who dropped out. Table 3 shows the number of participants reporting each symptom, including additional symptoms they self-reported. The only symptoms that were significantly different between MAD and betaquik^®^ were abdominal pain occurring more often with betaquik^®^ at the 3-month time point and nausea occurring more often with betaquik^®^ at the 6-month time point. Note that participants who dropped out prior to each time point did not complete the 3- and/or 6-month surveys, and all participants who dropped out reported gastrointestinal symptoms. Fewer symptoms were reported for both MAD and betaquik^®^ at the 6-month time point compared to the 3-month time point. There was 1 less participant at the 6-month time point, but even if the symptoms they reported at 3-months were added to the 6-month time point, there would be a total of 23 symptoms reported at 3-months and 18 at 6-months.

## 4. Discussion

There was 100% adherence for consuming betaquik^®^ added to MAD for 10 days each month in the 13 participants who completed study, and also in the three participants who dropped out. The overall intervention adherence rate (considering the three participants that dropped out) through study completion was 81.2%. This rate of adherence to betaquik^®^ is better than reported for MAD alone in comparable studies over 6 months, although all participants in this study were pre-screened for MAD adherence and were motivated to try an intervention for additional seizure reduction, which may account for this finding. Monthly contact with participants and knowing they would be contacted for a food recall every month was likely motivating and helped with adherence. Rates may be lower if patients are not monitored as closely.

Participants reported that betaquik^®^ added to MAD was more convenient and easy to use compared to MAD alone. This is somewhat surprising as it was an additional intervention they needed to incorporate for 10 days per month, but is a reflection of the general ease of taking the product. Participants did not like the taste of betaquik^®^ compared to MAD alone, but this difference was not significant.

Overall tolerance of betaquik^®^ compared to MAD was very good, although 3 of 16 study participants dropped out due to gastrointestinal side effects that were not present prior to initiating the intervention. The most common side effects attributed to MAD alone were constipation and nausea, whereas the most common side effects attributed to betaquik^®^ were abdominal pain, diarrhea, and nausea. An attempt was made to ease side effects and improve tolerability by having participants consume a smaller amount on the first of 10 days, and they also had the option of spreading their total consumption throughout each day instead of consuming it all at once. In the future, starting with even smaller amounts may help with tolerance or consistently using a small amount daily and increasing during the 10-day catamenial period. Additionally, customized instructions (i.e., amount and frequency) could be provided based on an individual’s prior experience with MCT products. For example, if a participant previously tried MCT oil as a ketogenic supplement and had GI side effects, starting with a lower dose over a longer titration period and/or implementing a slower titration during the 10 days and/or consuming over divided doses throughout the day may help ease or prevent side effects. The 10-day timeframe for the intervention was chosen as standard duration to target catamenial seizure patterns, but the timeframe could be extended to allow for titration. Participants may have become more accustomed to MCT oil over time, and this is reflected in the symptom survey, which showed fewer symptoms reported for betaquik^®^ at the 6-month time point compared to the 3-month time point, even when taking into consideration symptoms reported by participants who dropped out of the study.

Interestingly, 94% of study participants primarily had a C3 (luteal phase of the menstrual cycle) catamenial pattern. This finding was consistent with progesterone levels, which were suggestive of anovulation for 64% of the participants. The C3 catamenial seizure pattern can be particularly difficult to treat because there are typically anovulatory cycles with irregular (and thus difficult to predict) menses and cycle lengths. For example, it is very difficult to accurately time an intervention for a woman with increased seizures 1 week prior to menses if menses are irregular. This was a challenge during the present study because a 10-day time frame that would have captured the time of increased seizures prior to menses for 1 month may not have correlated to the same phase of the cycle during the next month. In some cases, a different 10-day window had to be provided each month (i.e., we were not always able to say “start consuming betaquik^®^ on day 10 of menses every month”). Outside the context of a research study, determining the exact timing for multiple patients would be difficult to coordinate in a busy clinical practice. Further studies are needed to determine whether the C3 catamenial pattern is seen more often in women on MAD and if MAD itself could be contributing to anovulatory cycles, irregular menses, and lower than expected progesterone levels.

Although the catamenial seizure pattern did not typically change to a different catamenial pattern from month to month, 75% of participants had some months during the intervention where there were seizures but no identified catamenial pattern. Notably, 37.5% had at least one intervention month with no seizures. Overall, 43.7% had reduced seizures, including 25% with a 50% or greater seizure reduction, during the intervention months compared to the baseline month. One participant had six seizures during the baseline month and six total seizures during the 5 intervention months combined.

Most women did not consistently record their ketone levels during the study, despite being provided with urine ketone test strips. This is not necessarily surprising because it can be a challenge for patients on MAD seen in the AEDC and AEDTC to consistently monitor, record, and self-report ketone levels. In this study, participants were asked to record ketone levels more often than typically requested in clinical practice. However, for participants who did record ketones, 68.8% had higher ketones compared during the 10-day window on 1 or more of the months betaquik^®^ was used, compared to the baseline month. The goal of using betaquik^®^ is to boost ketone body production by increasing MCT fat intake (25 g for 9 of the 10 intervention days), which may result in higher than baseline ketones or maintaining ketone levels during times when the ketone levels may be lower. For example, some women note a trend towards lower ketones during menses compared to the rest of the month.

Limitations of the study included its small sample size and that the enrollment goal was not met. Recruitment for the study proved more challenging than anticipated. All women who qualified for the study chose to enter the study, but finding women on MAD who met all of the enrollment criteria was challenging. Of note, in our clinical experience, many women self-report catamenial seizure patterns even if no consistent patterns are identified on calendar review. In addition, some women who reported catamenial seizure patterns in clinic were not able to provide seizure and menstrual cycle data for the minimum three full menstrual cycles that needed to be analyzed to determine if there is a catamenial pattern.

Future considerations include a larger clinical trial to evaluate interventions and strategies for catamenial seizure patterns in women on KDTs. The adherence rate and tolerability of betaquik^®^ shows feasibility for future studies. There is a great need to develop better treatments for catamenial epilepsy in general, but in particular for women with a C3 catamenial pattern. A future study could evaluate whether consuming betaquik^®^ (or equivalent) consistently (instead of for 10 days per month) would be most effective in eliminating seizures in a C3 pattern.

## Figures and Tables

**Table 1:** Participant demographics and epilepsy characteristics.

	Enrolled in Study (*N* = 16)	Completed Study (*N* = 13)
Mean age, y (SD)	32.8 (8.0)	30.6 (6.3)
Epilepsy classification, *n* (%)		
Focal	10 (62.5)	9 (69.2)
Generalized	5 (31.3)	3 (23.1)
Generalized and Focal	1 (6.3)	1 (7.7)
Mean age of onset of seizures, y (SD)	10.9 (10.8)	8.0 (7.3)
Mean duration of epilepsy, y (SD)	20.1 (11.8)	20.3 (11.4)
Median baseline seizure frequency/week, (IQR)	1.79 (4.2)	1.58 (3.0)
Mean age at KDT start, y (SD)	30.9 (8.5)	28.3 (6.5)
Intellectual disability, *n* (%)	2 (12.5)	2 (15.4)

y = years; SD = standard deviation; KDT = ketogenic diet therapy; IQR = interquartile range.

**Table 2:** Tolerance.

	Baseline-MAD Only (*n* = 16)	3 Month-MAD + Betaquik^®^ (*n* = 14)	6 Month-MAD + Betaquik^®^ (*n* = 13)
Convenience (SD, *p*-value *)	6.5 (2.6)	7.71 (2.6, **0.043**)	8.15 (2.1, **0.025**)
Ease of use (SD, *p*-value *)	7.25 (2.4)	8.07 (2.6, 0.19)	8.77 (1.8, **0.014**)
Taste (SD, *p*-value *)	7.88 (2.3)	7.36 (2.8, 0.784)	7.54 (3.0, 0.602)
Average on a 1–10 Likert scale with 1 = worst and 10 = best			

* *t*-test compared with baseline, MAD = modified Atkis Diet, SD = standard deviation, bold = significant.

**Table 3:** Symptoms reported by participants attributed to MAD or betaquik^®^.

	Baseline-MAD Only (*n* = 16)	3-Month MAD (*n* = 14)	3-Month Betaquik^®^ (*n* = 14)	6-Month MAD (*n* = 13)	6-Month Betaquik^®^ (*n* = 13)
Abdominal pain, *n* (%)	3 (18.8)	0	5 (31.3) *	0	3 (23.1)
Constipation, *n* (%)	6 (37.5)	3 (21.4)	1 (7.1)	4 (30.8)	1 (7.7)
Diarrhea, *n* (%)	2 (12.5)	1 (7.1)	4 (28.6)	0	2 (15.4)
Nausea, *n* (%)	4 (25)	1 (7.1)	4 (28.6)	1 (7.7)	5 (38.5) *
Vomiting, *n* (%)	1 (6.3)	0	0	0	0
Fatigue, *n* (%)	2 (12.5)	1 (7.1)	0	0	0
Hunger, *n* (%)	2 (12.5)	2 (14.3)	2 (14.3)	0	0
Problem with texture/mouthfeel, *n* (%)	0	0	2 (14.3)	0	1 (7.7)
Problems with taste, *n* (%)	0	1 (7.1)	2 (14.3)	1 (7.7)	1 (7.7)
Additional symptoms, *n* (%)	4 (25) ^a^	2 (14.3) ^b^	3 (21.4) ^c^	0	1 (7.7) ^d^

* *p*-value < 0.05 when comparing MAD and betaquik^®^ at the 3-month and 6-month time points, ^a^ hematuria, increased seizures, rash around mouth, sensitivities to certain foods, ^b^ hematuria, weight loss, ^c^ small seizures worse, has to drink slowly else has abdominal pain, rash on roof of mouth, ^d^ bloating and cramping, MAD = modified Atkins diet.

## Data Availability

The data presented in this study are available on request from the corresponding author. The data are not publicly available due to privacy.
